# The Latin American Legislators Dataset

**DOI:** 10.1038/s41597-025-05882-0

**Published:** 2025-11-11

**Authors:** Michael Weiss, Karel Kouba

**Affiliations:** Charles University, Center for Ibero-American Studies, Faculty of Arts, Prague, Czech Republic

**Keywords:** Politics, Government

## Abstract

This article describes a dataset of legislators in Latin America. It is a large data collection effort containing data on all elected legislators in the 18 Latin American countries with competitive elections since 1978 or transition to democracy up until 2023. The Latin American Legislators Dataset includes data both on unicameral legislatures as well as on the lower and upper chambers in bicameral legislatures and currently comprises 31,724 observations of 21,807 unique legislators in 259 elections. The dataset is an important resource for the study of legislative elites because it comprises information on various legislator attributes (esp. gender, elite turnover, seniority and inter-chamber switching).

## Background & Summary

We present a dataset containing information on 31,724 observations of 21,807 unique legislators in 259 elections in all 18 Latin American countries that have held competitive parliamentary elections since 1978 (or transition to democracy) up until 2023. The Latin American Legislators Dataset (LALD) identifies the names of all elected legislators in both upper and lower (or only) chambers together with variables describing their individual characteristics such as sex and party affiliation, basic features of the constituency for which they were elected, and election-related characteristics such as election date and whether it concerned a regular or snap election. It contributes (1) by providing the first cross-national database of national legislators with a concise coverage and a long time span in a global region that has not received scholarly attention compared to Western Europe and the U.S., (2) by identifying unique legislators which allows to trace their legislative presence over time in a single chamber as well as their movement across legislative chambers, lending itself to various operationalizations of legislature professionalization, (3) by including information from both lower and upper chambers, and (4) by identifying electoral districts at the individual legislator level, which allows for a more accurate measurement of institutional effects in quantitative analyses. The dataset will serve researchers in important research agendas that require knowledge regarding the composition of legislatures. These include examinations of the descriptive representation of women^1,2^ agendas involving legislators’ careers, especially those analyzing legislative turnover^3,4^, legislator reelection^5^, chamber switching^6^ and legislator professionalization^7^, as well as studies focused on institutional aspects of legislator behavior.

The main contribution of this dataset is to present a large and easily accessible consolidated dataset with a continental coverage and a large time span. This is an improvement over existing research and a contribution to the literature in four ways. First, most existing legislator datasets are limited to individual countries. The advantage of focusing on individual countries lies in providing a long time span of available data, stretching as far as the beginning of electoral politics since the 19th century until today in uninterrupted time series, as is the case of datasets on Denmark^8^ and the United Kingdom^9^. Post-WW2 legislator data with abundant biographical and contextual information is also covered for Germany^10^, as well as in a three-country comparison of Germany, Netherlands, and Switzerland^11^. Similar single-country datasets include data on provincial legislatures in Canadian provinces^12^, the Czech Political Candidate and Donation Datasets^13^, as well as electoral data on Spain^14^, the U.S.^15,16^, and Turkey^17^.

Second, to the extent that legislator datasets are comparative and include a larger number of countries, they are either static^18–21^, limited in the time span^22^, or do not provide a comprehensive regional coverage but focus on selected countries only^22–24^. The Comparative Legislators Database^24^, while featuring a long time span and data on important political and occupational characteristics, covers over 67,000 legislators in 16 countries located on 4 different continents. The Global Legislators Database^22^ includes many more countries, 97 in total, but its temporal coverage focuses on a single temporal point between 2015 and 2017, and also does not comprehensively cover any single global region. Similarly the Global Leadership Class^21^ dataset provides a static picture of legislators at a single point in time between 2010 and 2013 in 145 nation-states and is complemented with a wealth of biographical data. Other cross-national datasets providing a single-election static perspective have also been compiled for different analytical purposes^18–20^. Where comparative data efforts cover longer time spans in multiple countries (and sometimes go as far as collecting information on electoral rules and candidate lists^25^ or collecting individual MP career information such as for the Members of the European Parliament^26^), they never extend beyond Europe^27^, leaving out other world regions.

The state of the art therefore suggests that efforts to collect and present legislator datasets face tradeoffs, as it is unrealistic to simultaneously maximize all convenient features. The most important tradeoff is between the *breadth* of the dataset, in terms of the temporal coverage or the number of countries, and its *depth*, in terms of the number of variables describing the legislators. The Latin American Legislators Dataset is situated at the side of the tradeoff which maximizes breadth over depth. Unlike the CLD and GLD datasets, it does not feature information on legislators’ previous occupations or education^22,24^, as these data are not commonly included in electoral sources. However, sacrificing analytical depth comes with two crucial benefits. First, the LALD provides the first region-wide comparative dataset of national legislators with a full coverage of countries within a global region. Second, it includes a long time span covering the periods since the onset of the third wave of democracy. Furthermore, while we recognize the limitations in terms of depth, we try to remedy this by extracting as much data from electoral sources as possible. Additionally, we also utilize various methods to identify returning legislators across various elections, giving the dataset as much depth as possible within the confines of its design.

Third, the LALD is also unique in providing information on both lower and upper chambers. Existing comparative datasets usually only cover lower chambers. While lower chambers are undoubtedly important, the upper chambers also play a crucial role in bicameral legislatures and should therefore not be disregarded^28^. Moreover, their existence also provides additional political offices that legislators can strive for to extend their political careers. This has consequences for their political experience and legislative professionalization.

Fourth, one limitation for the study of legislators in Latin America is the nature of political data conservation in the region. While most 21st century elections have dedicated websites, these are - like websites of electoral bodies - often region-blocked, not allowing access to researchers across the globe, but rather only to those in certain countries. While the region-block can be circumvented, these websites often cease to be accessible after some time or lose their functionality due to current-day internet browsers no longer supporting previous website technologies for safety concerns. Due to old election websites not being updated to renew the accessibility of data, preserving legislator data in an accessible format is an important end in itself.

## Methods

The data was collected from primary sources, principally from elected legislator lists published by official electoral bodies. We reverted to secondary sources only when primary sources were not available. In cases where data was available in downloadable or scrapable formats, we used these to ease data collection for the given election. However, in many cases data was only available in the form of scanned documents, leading us to manually code legislator lists. In such cases the data for the election was double-checked to ensure its accuracy. The sources of the data are listed in the Online Appendix at the Open Science Framework (OSF)^29^ together with the dataset. We list both official sources and, where necessary, also secondary sources, for each election in each country.

After lists of elected legislators from all the sources were collected, we standardized the layout of the data to unify it across all countries. A sequential double-digit ID was created for each country. Using various techniques (for more information see the section on technical validation) we identified returning legislators and assigned them a unique temporary legislator ID. This ID variable was used in combination with the variable *CountryID* to create *LegislatorID*, a 7-digit variable that is both unique to each legislator and reflects the country in which the legislator was elected. We further created the *ObservationID* variable that uses *LegislatorID*, but adds the *ElectionYear* to the end of this variable, creating an 11-digit variable for each unique observation of a legislator being elected. This way of coding the country and election year into each observation allows for the unique ID to reflect these variables and allows for an easy identification of country and year within the variable, with no need to identify it in the respective fields.

Election-specific information was complemented with information concerning whether a legislator is male or female (based on legislators’ names) as well as about the exact election date^30^, and, if applicable, also about the date of the previous election^30^, a binary snap election variable^30^, and a binary variable differentiating between lower and upper chambers. Sources on election dates and snap elections are listed in the Online Appendix. Furthermore, using the exact election date allows for an easy measurement of term length in days, a more precise measure than term length in years.

Using a script in STATA, we created five variables that account for various types of legislator experience based on the variables identifying legislators, observations, types of legislative chambers, as well as election year and the year of previous election to the given chamber. The first constructed variable is the variable *ParliamentaryTerms*, which counts the number of legislative terms the legislator has been previously elected to at the time of election, counting both parliamentary chambers in bicameral countries. The variable *ChamberTerms* counts the times the legislator has been previously elected to the same chamber at the time of election. In countries with unicameral parliaments both values are the same. Third, we constructed the variable *ImmediateReelection*, which is a binary variable indicating whether a legislator was elected to the same chamber in the previous election. The fourth of these variables is *ConsecutiveTerms* which counts how many times a legislator was consecutively elected to the same legislative chamber. Lastly, we created the binary variable *ChamberSwitch*. This variable indicates a situation where a legislator was also elected in the previous election, but not to the same chamber. Naturally, this variable has the value of 0 for all unicameral parliaments.

## Data Records

The dataset is freely available at the OSF^29^. The dataset contains variables at three levels (Table 1). First, the individual-level variables pertain to each legislator. These are the variables for a legislator’s name, sex, legislator ID, observation ID, a legislator’s election being invalidated, and variables accounting for political experience measured as the number of parliamentary terms served, the number of terms served in the specific parliamentary chamber, the number of consecutive terms served in the respective chamber, whether or not the legislator switched the parliamentary chamber since the last election, and if the legislator has been reelected immediately following the end of their previous parliamentary term.

**Table 1:** Variables included in the dataset.

Variable name	Description
Country	Name of the country
CountryID	ID of the country
ElectionDate	Date of the election (day-month-year)
ElectionYear	Year of the election
PreviousElectionDate	Date of the preceding election if applicable (day-month-year)
PreviousElectionYear	Year of the preceding election (if applicable)
NextElectionDate	Date of the succeeding election if applicable (day-month-year)
NextElectionYear	Year of the succeeding election (if applicable)
DaysSincePreviousElection	Days between ElectionDate and PreviousElectionDate
YearsSincePreviousElection	Years between ElectionYear and PreviousElectionYear
SnapElection	Indicates if the election was a snap election (yes = 1; no = 0)
District	Name of the electoral district
DistrictMagnitude	District magnitude of the electoral district
DistrictType	Indicates if the district was a single-member or multi-member district
Party	Name of the legislator’s political party
Coalition	Names all parties of the legislator’s coalition. Only used for Brazil
Name	Legislator’s name
Female	Legislator’s gender (female = 1; male = 0)
Invalid	Legislator’s election was invalidated (yes = 1; no = 0)
LegislatorID	7-digit ID unique to each legislator
ObservationID	11-digit ID unique to each observation
ChamberType	Parliamentary chamber (upper = 1; lower or only = 0)
ParliamentaryTerms	Number of terms the legislator has previously been elected for
ChamberTerms	Number of terms in current chamber the legislator has previously been elected for
ConsecutiveTerms	Number of consecutive terms a legislator has been elected for to the current chamber
ChamberSwitch	Variable indicating if the legislator switched chambers after the end of their previous parliamentary term (yes = 1; no = 0)
ImmediateReelection	Variable indicating if the legislator was elected to the current chamber in the previous election (yes = 1; no = 0)

Second, at the election-level, the dataset contains variables that are associated with several national-level characteristics of the election within which each legislator was elected. These variables are the name of the country, the ID of the country, the date of the election, the date of the previous election, the year of the election, the year of the previous election, chamber type, which differentiates between lower and upper chambers, and whether the election was a snap election or whether it was held in a previously scheduled regular interval.

Third, at the meso-level, LALD includes variables situated between the individual and the election level. These are the district magnitude (measured as a count variable of the number of legislators elected in the constituency), district-type (single-member or multi-member), district name, and the name of the party for which each legislator was elected. In the case of Brazilian elections, all parties constituting an electoral coalition that supported the legislator as a candidate are also listed.

The LALD contains complete legislator data during the observed period starting from 1978 or transition to democracy, up until 2023 for the following countries: Argentina^31–37^, Bolivia^38–41^, Brazil^42–44^, Chile^45^, Colombia^46–48^, Costa Rica^49–60^, Dominican Republic^61–69^, Ecuador^70–82^, El Salvador^83–93^, Guatemala^94–104^, Honduras^105–108^, Mexico^109–113^, Nicaragua^114–120^, Panama^121,122^, Paraguay^123^, Peru^124^, Uruguay^125^, Venezuela^126–132^. Some elections are missing from the dataset (Table 2). These omissions are due to a lack of reliable official information especially on early post-transition legislators and detailed electoral data. The following periods are missing from the dataset, totaling 15 elections that fall within the scope of the studied time range: Colombia 1978–1990, Honduras 1991–1993, Paraguay 1989–1993 and Venezuela 1978–1998. As we plan to update the LALD with future elections, we will also include the missing historical elections into the dataset which will require extensive archival research in individual countries.

**Table 2:** Countries, elections and observations of legislators included in the dataset.

Country	Years available	Number of elections	Number of observations	Number of unique legislators
Argentina	1983–2023	33	3,157	2,302
Bolivia	1980–2020	22	1,763	1,394
Brazil	1990–2022	16	3,946	2,051
Chile	1989–2021	18	1,352	618
Colombia	1991–2022	17	2,279	1,371
Costa Rica	1978–2022	12	669	609
Dominican Republic	1994–2020	14	1,384	842
Ecuador	1979–2023	16	1,547	1,151
El Salvador	1985–2021	13	1,044	549
Guatemala	1985–2023	11	1,441	926
Honduras	1997–2021	7	901	596
Mexico	1988–2021	20	6,736	5,469
Nicaragua	1990–2021	7	630	432
Panama	1994–2019	6	434	284
Paraguay	1998–2023	12	750	508
Peru	1980–2021	14	1,718	1,364
Uruguay	1984–2019	16	1,032	593
Venezuela	2000–2020	5	941	748
Total	1978–2023	259	31,724	21,807

Furthermore, there are three instances of legislator observations without names in the dataset. These are coded as invalid observations. The reasons for the invalidation within our data include the primary sources providing no data, a candidate being subsequently invalidated by an electoral body and not being listed by their name by the primary source or the party winning the seat not fielding enough candidates to fill the seat. While these three observations could also be simply left out of the dataset, we believe that their inclusion helps in keeping the correct number of legislators for a chamber. Another reason for the missing electoral data is due to insufficient data provided by primary sources. This pertains to the 1994 and 1998 Colombian elections for which we were unable to locate complete legislator lists. In total 5 legislators from the Senate and 20 legislators from the Chamber of Representatives are missing in the primary data provided by the Colombian National Electoral Council for 1994, and 2 senators for the 1998 elections.

To be able to trace back any possible coding errors and remedy them, the LALD keeps legislators’ names intact as they appear in the original data source. This often leads to retaining multiple different variants of the same name across various elections, given that the data sources sometimes use different name versions, for example using only one first and last name in one election, while listing a legislator’s full name in another election. However, this does not imply that the legislator is doubly counted. The dataset accounts for different variants of names through retaining a unique LegislatorID across all those observations identified as belonging to the same legislator, while listing the legislator’s name that was used in the primary source. One example of different names appearing is the Argentinian legislator listed as Guillermo Eugenio Mario Snopek for the Senate in 2017, who is listed as Guillermo Snopek for the Chamber of Deputies in 2015 and 2023.

## Technical Validation

We use three tools for validating the accuracy of the data contained in the LALD, addressing three potential problems. First, a potential concern with data accuracy involves correctly identifying a unique legislator from data records across different time periods and chambers. This is a crucial step that allows tracking the previous legislative experience of the legislator. As this variable was manually coded, two types of errors could have been committed. While the “type I error” involves falsely identifying two different legislators as being the same legislator, the “type II error” involves falsely identifying the same legislator as being two different legislators. These errors might arise due to different variants of names in electoral sources (e.g. some legislators’ names are recorded only using one first name and one surname, while in other records their two first names and two surnames are used). To reduce both type I and type II errors to the largest extent possible, we utilize three different checks. First, we removed all special characters present in legislators’ names. This nullifies the name variance caused by the (non-)usage of special characters by data sources. Second, we utilize secondary sources, such as websites of legislatures, news sites and Wikipedia, in order to cross-validate a legislator’s identity and full scope of terms in office of both lower and upper chambers. Third, due to legislators not always being listed under their full names, we also checked for repeated observations of the same legislators using the MS Excel function of fuzzy matching to identify similar names, following this practice already established by similar research^133,134^. Observations identified as possibly being related to the same legislators were manually checked and verified to ensure reliability of the data. In terms of fuzzy matching specifications, we kept the default similarity criterion of 0.8, resulting in a list of all names that exhibited a similarity at the threshold of 0.8, which were subsequently manually checked to identify if both names indeed belonged to the same legislator. The combination of these checks greatly increased the accuracy of identifying and matching legislators across elections, as the existence of both typographical errors and different ways of naming a legislator across elections is otherwise a much bigger hindrance to correct legislator identification.

**Fig. 1:**
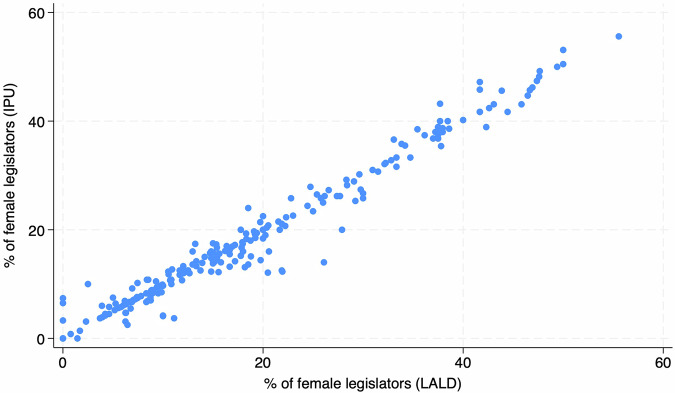
Relationship between LALD and IPU shares (in %) of female legislators.

The second validation is based on a comparison of LALD data on one of the manually coded variables - legislator gender - with an established aggregate-level database of gender representation in parliaments worldwide. We calculate the gender share based on the the number of female legislators elected to a parliamentary term at the time of an election. Changes to the composition during a parliamentary term are not captured by the LALD dataset and are therefore also not included in our gender share measure. The International Parliamentary Union (IPU) collects data provided directly by individual countries about the gender composition of parliaments^135^. While other datasets are also available, the IPU dataset provides a long historical coverage and importantly also includes information on both lower and upper chambers. Some legislative terms included in LALD are missing from the IPU data. However, the correlation between the share of female MPs in LALD with that of the IPU is near perfect with Pearson correlation coefficient *r* = 0.98 (see Fig. 1). This strongly suggests that the LALD data are accurate and reliable in terms of identifying male and female MPs. Additional checks were conducted on the elections that presented the largest differences between the LALD and IPU data on the share of elected female MPs. No errors were found in the LALD, leading us to assume that IPU data are based not on the share of elected female MPs, but rather on the share of female MPs present in parliament at the time of data collection. These figures naturally differ because legislators may exit during their term, affecting the legislature’s composition.

**Fig. 2:**
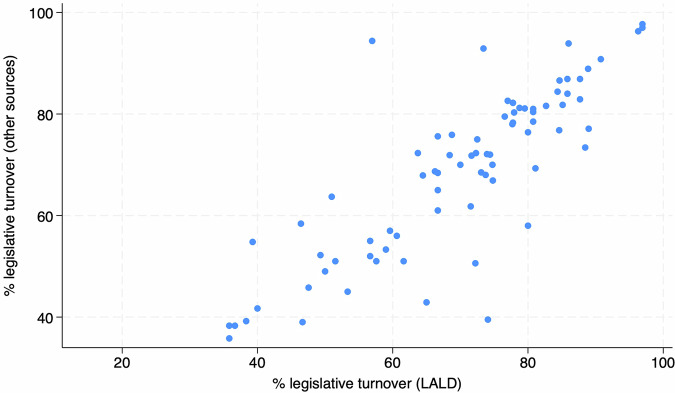
Relationship between LALD and secondary sources turnover rates (in %).

Third, we calculated legislative turnover from the LALD and compared it to turnover data from other sources^68,73,106,136–152^. Legislative turnover measures the percentage change of individual legislators from one election to the next and is commonly calculated as the percentage of legislators (of the total of elected legislators) elected to the chamber who were not elected in the immediately preceding election^3,4,146–151,153–159^. Due to focusing on elected legislators, this measurement includes only legislators who entered the chamber as a result of the election. It therefore excludes legislators entering the chamber later on during a legislative term (for example, as a result of resignation, death or other causes of early exit by elected legislators). Similarly, in cases of staggered elections, only legislative seats up for election are used to calculate legislative turnover. Unlike data on women’s legislative representation, there is currently no single legislative turnover dataset available for Latin American countries. We therefore used various secondary sources^68,73,106,136–152^ of legislative turnover and reelection rates to create a variable which was then compared to turnover rates calculated based on the LALD. Searching through secondary literature, we were able to identify legislative turnover rates from other sources for only 77 of the elections for which turnover rates can be calculated using the LALD. This is due to data unavailability, in part caused by upper chambers being mostly excluded from legislative turnover calculations. As captured in Fig. 2, several discrepancies between LALD data and these secondary sources are evident although the general pattern confirms a strong relationship between them, with the correlation between the LALD turnover and turnover collected from other sources being *r = *0.83. As legislative turnover studies do not commonly share the source data that legislative turnover is calculated from, the causes of the discrepancies may only be inferred. However, as authors often find very different rates of legislative turnover, these may be due to incorrect identification of repeating legislators across various sets of elections. Second, they may also be due to different ways that legislative turnover is calculated. Some studies do not explicitly state the way legislative turnover is operationalized, leaving the reader to infer the way it is calculated^159^, while others either use or mention alternative ways of calculating turnover such as relating to all legislators who have been members of parliament^154^, calculating turnover as changes within sessions of the European Parliament^160^ or calculating yearly turnover^3,161^.

While discrepancies between secondary sources on legislative turnover are often only within a few percentage points, they can sometimes be greater, even by as much as 10 points are more, with one source reporting legislative turnover for the Brazilian chamber of deputies in the 2002 elections of 39.6%^146^, while a second one of 45.8%^138^, with other authors reporting 49.5%^162^. Our own calculations show a turnover rate of 47.6%. Despite being illustrative, this example highlights the need for both transparent calculations and primary data being used to identify differences and possible errors. The largest discrepancies that we identified within our data are eight instances of LALD finding legislative turnover lower by over 10 percentage points and five instances of turnover higher by over 10 percentage points. The former can be explained by a better identification of legislators across elections thanks to using various techniques to match recurring legislators. The latter, however, is more difficult to explain without being able to see the source data of other sources. The four prominent cases of LALD data reporting higher turnover rates than other sources are Senate elections in Brazil in 2002 and 2006, in Chile in 2005, and in Uruguay in 1989. Except Uruguay, these are all staggered elections. However, other staggered elections such as the Chilean senatorial elections in 2001, and most of the Argentinian elections except the lower chamber elections in 1993, 1995, and 2001, show similar turnover rates. It is therefore possible that some sources calculated legislative turnover in staggered elections differently. There is usually little to no explanation of how legislative turnover was calculated in existing sources of turnover data. Therefore, reporting very different legislative turnover rates despite also using some of the same sources for legislative turnover on primary legislator data^69,74,107,131,136–144^ leads us to believe that either different calculations were used (such as including within-session turnover), or that other sources identified subsequent legislators differently.

## Usage Notes

The LALD serves as a resource for several important research agendas concerning legislative politics in Latin America. Three such agendas are especially pertinent. First, the LALD allows for the calculation of various indices of parliamentary elite turnover, renewal, reelection and overall professionalization. Because this is the first legislator-level dataset with a long temporal coverage including all countries in Latin America, its data may fuel the analyses of both the causal determinants of legislative turnover, as well as of its consequences. These issues have raised great scholarly interest in other (mostly European) countries^3,157,163^. Surprisingly, a single comparative treatment on the legislature-level has been performed in Latin America, suggesting a great potential of the dataset^4^. Additionally, given the wide temporal range of the LALD dataset for some countries, it is possible for the first time to differentiate between first-term and returning MPs in measures of legislative turnover. The need for such applications of turnover measures has already been discussed by other researchers^156,164^, but these have to our knowledge not been applied. While not explicitly mentioned, the lack of long-term data for legislator careers is most likely the leading cause for research not differentiating first-term and returning MPs. Additionally, current research on legislative turnover does not commonly share individual-level legislator data used to calculate turnover rates. As part of our effort to contribute to open science, we believe the LALD to be a step in the right direction of making this type of source data available to researchers.

Second, the inclusion of another individual characteristic in LALD - legislator gender - may inspire more focused examinations of women’s descriptive representation in Latin American legislatures. Analyzing the determinants of women’s presence in legislatures has produced a number of insightful analyses with data aggregated at the level of each elected legislature^1,2^. Building on this agenda, LALD additionally provides opportunities to explore determinants of political gender representation at the individual level, potentially combining determinants set at the national level with those at the individual levels within a hierarchical data structure.

Third, district magnitude presents a crucial topic of electoral studies^165^. Accurately collecting all information on district magnitude across Latin American elections has been challenging and LALD can be a useful source in this respect. Due to the nature of the LALD being based on primary data sources and including district magnitude in almost all observed elections, it is a comprehensive source for district magnitude and its changes in Latin American countries with a wide temporal coverage.

## Data Availability

All replication code is available on the OSF^29^. All transformations of the dataset as well as figures in this data descriptor were performed using Stata (version 18.5). We include*.do* files with code for the replication of the following variables based on the variables LegislatorID, ObservationID and ChamberType: ParliamentaryTerms, ChamberTerms, ConsecutiveTerms, ChamberSwitch and ImmediateReelection. We also include replication code in the*.do* file format for the two validation measures of female legislator share and legislative turnover, using the LegislatorID variable alongside Female and ImmediateReelection, respectively.
